# Novel Polyomavirus associated with Brain Tumors in Free-Ranging Raccoons, Western United States

**DOI:** 10.3201/eid1901.121078

**Published:** 2013-01

**Authors:** Florante N. Dela Cruz, Federico Giannitti, Linlin Li, Leslie W. Woods, Luis Del Valle, Eric Delwart, Patricia A. Pesavento

**Affiliations:** Author affiliations: University of California, Davis, Davis, California, USA (F.N. Dela Cruz, Jr., F. Giannitti, L.W. Woods, P.A. Pesavento);; Blood Systems Research Institute, San Francisco, California, USA (L. Li, E. Delwart); Louisiana State University, New Orleans, Louisiana, USA (L. Del Valle);; University of California, San Francisco, San Francisco (E. Delwart)

**Keywords:** Polyomavirus, raccoon, oncogenesis, malignant peripheral nerve sheath tumor, glioblastoma, oncogenic virus, tumor virus, Merkel cell polyomavirus, JC virus, olfactory tract, California, large T-antigen, p53, viruses, United States

## Abstract

Tumors of any type are exceedingly rare in raccoons. High-grade brain tumors, consistently located in the frontal lobes and olfactory tracts, were detected in 10 raccoons during March 2010–May 2012 in California and Oregon, suggesting an emerging, infectious origin. We have identified a candidate etiologic agent, dubbed raccoon polyomavirus, that was present in the tumor tissue of all affected animals but not in tissues from 20 unaffected animals. Southern blot hybridization and rolling circle amplification showed the episomal viral genome in the tumors. The multifunctional nuclear protein large T-antigen was detectable by immunohistochemical analyses in a subset of neoplastic cells. Raccoon polyomavirus may contribute to the development of malignant brain tumors of raccoons.

The American Cancer Society estimates that infectious pathogens are associated with up to 20% of all human cancers worldwide. Among oncogenic viruses are those in the *Polyomaviridae* family, whose members infect an array of vertebrate species, including birds, humans, nonhuman primates, bovids, rodents, and sea lions (*1*,*2*). Infection in mammals typically results in persistent asymptomatic infection (*3*,*4*). However, natural disease studies of human infection and experimental disease studies suggest that a potential outcome of some polyomavirus (PyV) infections is tumor formation (*4*–*6*). Experimental evidence that PyVs are tumorigenic is 50 years old and not debated; PyV-induced tumorigenesis in laboratory animals, by simian virus 40 (SV40) or by multiple human PyVs, such as JC virus (JCV), is used extensively as a cell transformation model. JCV, for example, induces brain tumors when intracerebrally inoculated in experimental animals (*7*–*11*). Furthermore, transgenic mice harboring the viral-encoded large T-antigen (LT-Ag) alone develop tumors of neuroectodermal origin, including malignant peripheral nerve sheath tumors (MPNSTs) and glioblastomas. Evidence that PyVs induce tumors after natural infection is accumulating but more controversial. Studies reliant on molecular detection of tumor-associated virus in isolation, however extensive, are inconclusive because association between PyVs and naturally occurring neoplasms varies and because PyV infections are highly prevalent, yet tumor formation is rare (*3*,*4*,*12*–*14*). Thus, although PyV-induced oncogenesis in laboratory animals has been a prolific model for the study of the cell cycle and cell transformation, natural infections rarely result in tumor formation, so the steps in cell transformation after natural infection are being revealed more slowly. Recent advances have been made by an accumulation of studies on Merkel cell polyomavirus (MCPyV), which is highly associated and integrated in most Merkel cell carcinomas (*5*). However, several unanswered questions relating to persistence, transmission, and transformation remain.

Most veterinary diagnostic laboratories receive large numbers of raccoon (*Procyon lotor*) carcasses for diagnosis, yet tumors of any type are rarely reported (*15*–*17*). In northern California and southern Oregon, we diagnosed 10 cases of olfactory tract/frontal lobe brain tumors in free-ranging raccoons during March 2010–May 2012. During the same period, no other raccoon tumors were reported in diagnostic laboratories across the United States and Canada. The clustering of cases and association of the tumors with the olfactory pathways suggested an infectious cause with a distinct route of transmission and tropism. We characterized from these tumors a candidate etiologic agent, raccoon polyomavirus (RacPyV).

## Materials and Methods

### Tissue Samples

All tissues were obtained by necropsy from the California Animal Health and Food Safety Laboratory, Veterinary Medical Teaching Hospital, at the School of Veterinary Medicine, University of California, Davis, California, or the Veterinary Diagnostic Library of Oregon State University, Corvallis, Oregon. DNA was extracted by using the DNeasy Blood and Tissue Kit (QIAGEN, Valencia, CA, USA) according to the manufacturer’s instructions.

### Consensus PCR

Reactions were performed by using Phusion polymerase (New England Biolabs, Ipswich, MA, USA). Consensus PCR primers were designed on the basis of conserved stretches of amino acid sequences in multiple sequence alignments (Vector NTI, Invitrogen, Carlsbad, CA, USA) of LT-Ags from multiple PyV species. Primer sequences are listed in the Technical Appendix Table.

PCR conditions were as follows: 98°C for 10 sec, followed by 30 cycles of 98°C for 10 sec, 44°C for 40 sec, 72°C for 30 sec; then 72°C for 10 min; and a 4°C hold. Amplification products were subjected to electrophoresis on a 1% agarose gel, and amplicons were purified by using a QIAquick Gel Extraction Kit (QIAGEN).

### Sequencing Reactions

PCR products were Sanger sequenced with a BigDye Terminator v3.1 Cycle Sequencing Kit (Applied Biosystems, Foster City, CA, USA). This process was conducted on an ABI 3770 Genetic Analyzer (Applied Biosystems).

### Phylogenetic Analysis

Reference viral sequences from the following PyVs were obtained from GenBank: JCV (accession no. J02226.1), SV40 (J02400.1), MCPyV (JF813003.1), murine polyomavirus (J02288.1), gorilla polyomavirus (HQ385752.1), chimpanzee polyomavirus (2c/6413) (HQ385751.1), chimpanzee polyomavirus (Azzie) (FR692336.1), and orangutan polyomavirus (FN356901.1). Amino acid sequence alignments of the predicted RacPyV LT-Ag, small T-antigen (sT-Ag), viral protein (VP) 1, and VP2 were generated by using ClustalW, implemented in MEGA5 (www.megasoftware.net). Aligned sequences were used to generate phylogenetic trees in MEGA5 by using the neighbor-joining method with amino acid p-distances and 1,000 bootstrap replicates. Intron splice sites in RacPyV LT-Ag were predicted by using the NetGene2 server (www.cbs.dtu.dk/services/NetGene2/).

### Immunohistochemical Analysis

Tissue sections from the tumors and normal brain tissue from raccoons without tumors were sectioned at 4 μm thickness and placed on electromagnetically charged slides. Immunohistochemical analyses were performed by using the avidin-biotin peroxidase method as described (*18*). The primary antibodies used for this study included a mouse monoclonal antibody against a peptide from exon 2 of the MCPyV that specifically recognizes LT-Ag and 57-kDa isoforms but does not cross-react with sT-Ag (CM2B4; Santa Cruz Biotechnology, Santa Cruz, CA, USA) and a mouse monoclonal anti–wild-type p53 (Clone DO-7; Dako Laboratories, Carpinteria, CA, USA). Double labeling immunofluorescence was performed according to the protocol described above; however, secondary antibodies were tagged with Alexa-Fluor 488 for the LT-Ag primary and Alexa-Fluor 568 for the p53 primary (Invitrogen, Carlsbad, CA USA).

### Southern Blot Hybridizations

Genomic DNA from tumor and normal brain tissue was extracted by using the DNeasy Blood and Tissue Kit (QIAGEN) according to the manufacturer’s instructions. RacPyV_LT_Probe1, a 799-bp digoxygenin-labeled probe designed to hybridize to the RacPyV LT-Ag coding region, was PCR amplified from RacPyV DNA according to the manufacturer’s instructions by using the PCR DIG Probe Synthesis Kit (Roche, Indianapolis, IN, USA) and verified (Technical Appendix Figure 1). One microgram DNA undigested or digested with *Kpn*I at 37°C for 16 h was separated on a 0.8% agarose gel and transferred to positively charged nylon membranes (Roche) overnight by capillary action in 20× SSC. DNA was UV cross-linked and then hybridized with RacPyV_LT_Probe1 (Technical Appendix Figure 2). Hybridization occurred for 16 h at 48°C. Membranes were washed with washing buffer (0.1 mol/L maleic acid, 0.15 mol/L NaCl; pH 7.5; 0.3% [volume/volume] Tween 20) and developed with NBT/BCIP stock solution (Roche) for up to 2 h.

### Rolling Circle Amplification

Rolling circle amplification (RCA) was conducted by using the illustra TempliPhi 100 Amplification Kit (GE Healthcare Life Sciences, Piscataway, NJ, USA) according to the manufacturer’s instructions. Briefly, 1 μL DNA extracted from tumor or normal brain tissue was added to 5 μL sample buffer and incubated for 5 min at 95°C. The sample was cooled on ice and then combined with 5 μL reaction buffer and 0.2 μL enzyme mix. The reaction was then incubated at 30°C for 16 h, followed by incubation at 56°C for 10 min to deactivate the polymerase. RCA amplified products were *Kpn*I digested at 37°C for 16 h and separated on a 0.8% agarose gel.

## Results

### Clinical Findings and Tumor Pathology

**Figure 1:**
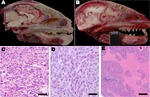
Pathology of raccoon polyomavirus–associated tumors. A) Normal anatomy, head of unaffected raccoon, midsagittal section. An intact cribriform plate separates the ethmoid turbinates from the olfactory tract. B) Gross pathology, head of raccoon no. 9 (Rac9), left parasagittal section. The tumor obliterates the left olfactory tract and extends into the left frontal lobe to the level of the midbrain. The tumor compresses the brain and distorts the cerebellum. The raccoon head in length (crown to nose) is 16 cm. C) Histopathology (hematoxylin and eosin staining) for Rac5. Marked anisokaryosis and anisocytosis are evident. Original magnification ×40. D) Histopathology (hematoxylin and eosin staining) for Rac10. Shown is a region that has streams of elongated cells. Original magnification ×40. Scale bar = 40 μm. E) Histopathology (hematoxylin and eosin staining) for Rac3. Cell-dense sheets of neoplastic cells are interrupted by vast regions of necrosis. Original magnification ×20.

During March 2010–May 2012, 52 raccoons were submitted to the California Animal Health and Food Safety Laboratory at the University of California, Davis, for full necropsy, with rabies screening performed as part of state diagnostic protocol. Ten (19%) raccoons had brain tumors within the cranial portion of their frontal lobe(s), most of which spanned the cribiform plate of the ethmoid bone and extended into, or from, the olfactory tract (Figure 1). Nine of the affected animals represented separate collection events from 3 adjacent counties in California, and 1 affected animal was shipped from southern Oregon. In all instances, the raccoons were exhibiting neurologic signs, including wandering in the daylight, approaching humans, or exhibiting lack of consciousness. Overall, other tissues, including lymphoid and hematopoietic tissues such as bone marrow, thymus, lymph nodes, and spleen, were generally within normal limits.

In one of the affected raccoons (Rac7), a presumed early (grossly undetectable) tumor was exclusively localized within the olfactory tract and segments of the axonal bundles of olfactory nerves within the ethmoid turbinates, which makes this the likely site of tumor origin. Most of the tumors were histologically pleomorphic, and each of the 10 tumors have been alternately and preliminarily classified as MPNSTs or glioblastomas on the basis of criteria such as anatomic location, histopathology (Figure 1), and a panel of histochemical and immunohistochemical stains, including reticulin stain, glial fibrillary acidic protein, vimentin, pancytokeratin, neuron-specific nuclear protein, Olig2, synaptophysin, and laminin. Among consistent findings in the glial tumors was expression of glial fibrillary acidic protein, variably sized regions of necrosis (Figure 1), and high mitotic activity. Tumors classified as MPNSTs also were positive for glial fibrillary acidic protein; however, neoplastic cells were individually surrounded by a reticulin-positive framework, which was immunoreactive with laminin. Viral inclusions were not detectable histologically, and no viral particles were visualized by transmission electron microscopy. 

### RacPyV Discovery by Consensus PCR

JCV is a human PyV that has been associated with brain tumors, including a broad range of glial-origin tumors. In experimental studies, transgenic mice expressing JCV LT-Ag under the control of the Mad-4 promoter develop MPNSTs and under the regulation of the CY (archetype) promoter develop medulloblastomas and glioblastomas (*19*). Because LT-Ag is a principal PyV protein that orchestrates oncogenesis (*13*,*20*–*25*), we chose it as the target for consensus PCR detection. Using an alignment of 21 PyVs, we designed 4 sets of degenerate primers to amplify conserved regions within LT-Ag (Technical Appendix Figure 3). Amplification was successful by using primers PLMA_F3 and PLMA_R1, yielding a 270-bp product from tumor tissue of the 3 raccoons originally tested. Thus far, the virus has not been detected in brain tissue or multiple other tissues (kidney, spleen, feces, salivary gland, lung, tonsil, nasal swab) tested in 20 unaffected animals collected from the same geographic region. Full length viral genomes (6/10) or partial genomes (4/10, ranging from 2,998 bp to 4,667 bp) were generated by using a combination of primer walking and specific RacPyV primers.

### RacPyV Genome Analysis

**Figure 2:**
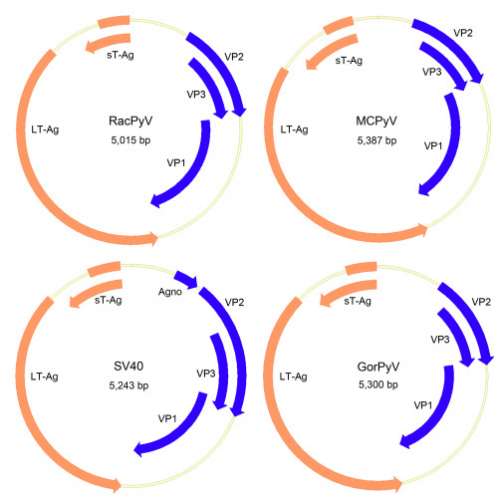
Genome organization of RacPyV. The entire dsDNA viral genome for RacPyV10 comprises 5,015 bp. The viral genome has a noncoding regulatory region and putative open reading frames for the late proteins VP1, VP2, and VP3 and the early proteins LT-Ag and sT-Ag. MCPyV and GorPyV, which are phylogenetic neighbors, and SV40 are presented for comparison. RacPyV, raccoon polyomavirus; LT-Ag, large T-antigen; sT-Ag, small T-antigen; VP, viral protein; MCPyV, Merkel cell polyomavirus; SV40, simian virus 40. GorPyV, gorilla polyomavirus.

The novel virus was most closely related, phylogenetically, to a clade of viruses that includes MCPyV and was named raccoon polyomavirus (RacPyV). PyVs are small, circular, double-stranded DNA viruses, and analysis of the RacPyV genome showed characteristic organization for *Polyomaviridae* (Figure 2) (*6*). RacPyV encodes putative open reading frames (ORFs) with homology to PyV LT-Ag, sT-Ag, the structural VPs 1–3, and a single bidirectional noncoding regulatory region (NCRR). Because of sequence variation among the raccoon isolates, the designations 1–10 indicate the order of animal submission and which raccoon harbored which respective RacPyV; that is, RacPyV1 was found in Rac1 and was the first tumor and RacPyV discovered.

**Figure 3:**
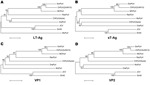
Phylogenetic relationship of RacPyV with representative polyomavirus species. Phylogenetic trees were individually generated on the basis of amino acid sequences of LT-Ag (A), sT-Ag (B), VP1, and VP2 (C, D) by using the neighbor-joining method with p-distance and 1,000 bootstrap replications. RacPyV, raccoon polyomavirus; LT-Ag, large T-antigen; sT-Ag, small T-antigen; VP, viral protein; GorPyV, gorilla polyomavirus; ChPyV, chimpanzee polymavirus; MCPyV, Merkel cell polyomavirus; OraPyV, orangutan polyomavirus; JCV, JC virus; SV40, simian virus 40; MuPyV, murine polyomavirus. The bar represents 5% estimated phylogenetic divergence.

**Figure 4:**
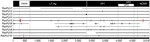
Partial genome sequences (ranging from 2,998 bp in raccoon polyomavirus 6 [RacPyV6] to 4,667 bp in RacPyV9) were obtained from 4 raccoons that either had undergone prolonged storage (RacPyV9) or were available only as formalin-fixed, paraffin-embedded tissue (dashed lines, RacPyVs 1, 6, and 7). Gaps in the sequences correspond to regions where amplification reactions failed. The genomes of RacPyVs 2, 3, 4, 5, 8, and 10 were sequenced in their entirety by using a primer walking method to complete the RacPyV circular genome. Open circles represent noncoding mutations; the closed circle represents a coding mutation. Horizontal bars indicate deletions. LT-Ag, large T-antigen; VP, viral protein; NCRR, noncoding regulatory region.

Phylogenetic trees of the deduced PyV proteins constructed by using the neighbor-joining method show that the putative early and late proteins of RacPyV are most closely related to those of MCPyV and chimpanzee PyV (Figure 3). The 10 RacPyV genomes had a total of 30 single nucleotide polymorphisms (SNPs) (Figure 4). Most (29/30) of the SNPs were synonymous substitutions or in noncoding regions, as seen with strain variants of other PyVs. Half (15/30) of the SNPs were unique to the single Oregon case (Rac6), perhaps reflecting its geographic distance from the California raccoons. Three distinct nucleotide deletion events occurred across RacPyV5 and RacPyV10. RacPyV5 had a 26-bp deletion within the putative LT-Ag intron and a 12-bp deletion within the putative NCRR; RacPyV10 had a 1-bp deletion within the putative LT-Ag intron. The genome of RacPyV3 is deposited in GenBank (accession no. JQ178241).

The NCRR of RacPyV encodes multiple consensus pentanucleotide LT-Ag binding sites (GAGGC) on both strands as seen in other PyVs. RacPyV LT-Ag has 46% homology to MCPyV LT-Ag and has features conserved among PyV LT-Ags, including the LXCXE motif necessary for LT-Ag association with the retinoblastoma protein and the Walker A box GPXXXGKT, which suggests that RacPyV LT-Ag is an ATPase that binds to p53 (*26*). In experimental models, these sites are crucial components of virus-mediated transformation (*20*,*22*,*23*).

The putative ORF encoding LT-Ag in RacPyV encodes putative splice sites surrounding a 387-bp intron. In other PyVs, sT-Ag, which shares a start site with LT-Ag, promotes cell transformation through negative regulation of the protein phosphatase 2A (PP2A) family of serine-threonine phosphatases (*26*,*27*). In transcription and expression studies, sT-Ag terminates at a stop codon within the LT-Ag intron. Aside from chimpanzee PyV (Azzie isolate) (*28*), all PyVs discovered thus far contain this canonical intronic stop codon that defines the ORF for sT-Ag. However, among the 7 RacPyVs for which the intron of LT-Ag was completely sequenced, only 2 (RacPyV5 and RacPyV10) contain intronic in-frame stop codons that would define an independent sT-Ag. In the 5 other RacPyVs, the entire T-antigen coding region is in-frame as a putative single ORF. Although expression studies are needed, among possible explanations are that a single T-Ag contains both LT-Ag and sT-Ag domains, that sT-Ag could be expressed as an alternately spliced form of the T-Ag coding region, that sT-Ag is generated proteolytically, or that it is not expressed. Of the 2 viruses that have a stop codon in-frame within the LT-Ag intron, RacPyV10 has a stop codon that terminates sT-Ag after the PP2A domains, as is seen in other PyVs, whereas RacPyV5 encodes a stop codon before the PP2A domains. However, the sT-Ag of MCPyV constructs altered to lack a functional PP2A binding domain still initiates cell transformation (*29*).

### Episomal RacPyV

**Figure 5:**
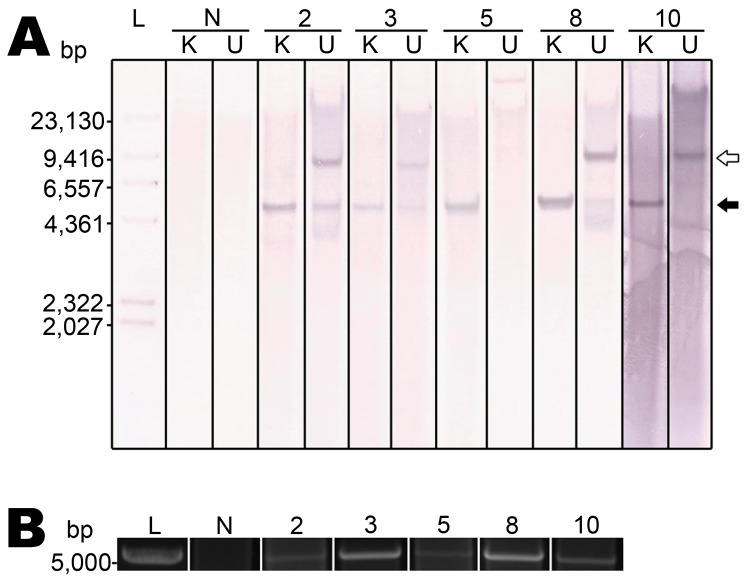
Raccoon polyomavirus (RacPyV) is episomal in raccoon brain tumors as detected by Southern blot hybridization and rolling circle amplification. A unique *Kpn*I site in viral protein 1 (Technical Appendix Figure 2) conserved across the viral genomes was predicted to linearize circular RacPyV DNA. Genomic DNA was *Kpn*I digested and probed with a 799-bp probe designed to hybridize to large T-antigen (Technical Appendix Figure 3). A) Southern blot hybridization. DNA digested with *Kpn*I (K) and undigested DNA (U) and hybridized with RacPyV_LT_Probe1 shows identical banding patterns in each tumor. In *Kpn*I-digested samples, a single band appears at ≈5 kb (closed arrow), which is the expected position for linearized RacPyV genome. B) RCA applied to RacPyV DNA by using random hexamers was digested with *Kpn*I. Amplification of the circular RacPyV genome occurred in the same cohort of samples that was successful for the Southern blot hybridization. L, DNA ladder; N, DNA extracted from a raccoon that did not have a brain tumor (i.e., normal raccoon).

Southern blot hybridizations and RCA were performed to ascertain whether RacPyV existed in episomal, integrated, or a combination of these forms. Viral DNA of sufficient quantity and quality was present in 5/10 tumors for these assays. In all cases, an ≈5-kb band for the *Kpn*I-digested samples was absent or present faintly in undigested conditions (Figure 5, panel A, closed arrow). The band variably present at ≈9 kb is expected to be a circular nicked form of the genome (Figure 5, panel A, open arrow). RCA, by using random hexamers, was performed on DNA extracted from the same set of tumor tissues. RCA products of the tumor tissues produced the expected ≈5-kb band after *Kpn*I digestion, confirming the Southern blot result that RacPyV is not detectably integrated in the host genome and demonstrating that it exists in circular form in tumor cells (Figure 5, panel B). As expected, Southern blot and RCA analyses performed on DNA extracted from normal, unaffected raccoon brain tissue produced no bands (Figure 5, panels A, B).

### Expression of RacPyV LT-Ag in the Nuclei of Tumor Cells

**Figure 6:**
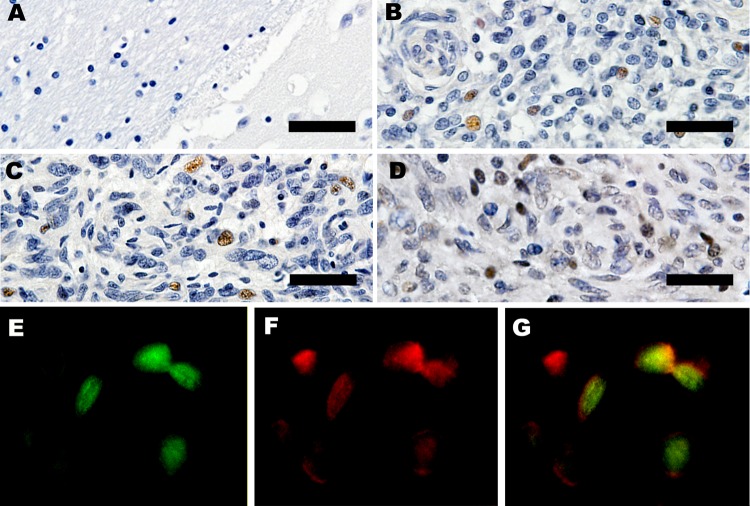
Expression of large T-antigen (LT-Ag) and p53 within a subset of tumors. A) Control, immunohistochemical analysis of frontal lobe of normal raccoon brain tissue. Astrocytes in this image and throughout the section were not immunoreactive for LT-Ag. Original magnification ×40. B, C) Immunohistochemical analysis for raccoon no. 2 (Rac2) and Rac3. LT-Ag is expressed within the nuclei of a subset of neoplastic astrocytes in 2 independent tumors. Original magnification ×40. D) Immunohistochemical analysis for Rac3. p53 is present within the nuclei of a subset of tumor cells. Original magnification ×40. Scale bars for panels A–D = 100 μm. E–G) Immunofluorescence of Rac4. p53 co-localizes with LT-Ag within nuclei of neoplastic cells. Original magnification ×60.

To determine the expression of LT-Ag, we performed immunohistochemical analyses with a monoclonal antibody that recognizes a peptide within exon 2 of MCPyV LT-Ag. The LT-Ag was absent (Figure 6) in unaffected regions of the brain and absent in unaffected raccoon tissues but was detected within the nuclei of a subset of neoplastic cells in 4 tested raccoon tumors (Figure 6). Immunohistochemical analyses for wild-type p53 also was positive in a subset of neoplastic cells (Figure 6) but was negative in the adjacent normal brain. Furthermore, double-labeling immunofluorescence showed the co-localization of LT-Ag with wild-type p53 in the nuclei of neoplastic cells (Figure 6).

## Discussion

We consider this an outbreak of brain tumors because a retrospective case search and literature review of ≈700 necropsies from California and other regions of the United States and Canada showed only 2 primary brain tumors in raccoons, both classified as low-grade astrocytomas (*30*,*31*). Because of the consistent anatomic location of the tumors, the absolute association with RacPyV, and the expression of LT-Ag, we propose that RacPyV might play a role in tumorigenesis. In our sampling of tissues from affected and unaffected raccoons, viral genomic sequences were limited to the neoplastic tissue of the brain and olfactory tract. A more extensive set of samples might uncover RacPyV viremia and shedding sites, but it is also worth considering that the raccoon might be a dead-end host for viral replication and that viral persistence and shedding might occur in a different species. Raccoons in the affected geographic region have a nomadic suburban lifestyle and use sewer and water lines for travel and presumed daily exposure to urine, garbage, environmental toxins, and environmental pathogens, including PyVs, which have been consistently detected in raw urban sewage samples worldwide (*32*).

Compared with other PyVs, RacPyV has several unique features. sT-Ag, for example, which in SV40 and other PyVs contributes to cell transformation (*27*,*29*,*33*), had a predicted termination site within the LT-Ag intron in only 2/7 RacPyVs analyzed. RacPyV has no predicted ORF or sequence homology for the Agno gene, which is found in many PyVs. RacPyV does not appear to be integrated into the host genome because both Southern blot hybridization and RCA analyses indicate that full-length genome exists in an episomal, circular state. Integration is one method of achieving replication incompetence of PyV and is considered by many researchers to precede cancer development (*34*). However, PyVs have alternate mechanisms of replication incompetence, and late gene suppression might occur in episomal virus. For example, Carbone et al. have implicated antisense RNA molecules produced in SV40 infection in late gene silencing through Dicer-mediated degradation (*35*). Natural and experimental infections with PyVs result in a spectrum of tumors that have some distinctions on the basis of route of infection, cofactors, and/or viral tropism (*7*,*8*,*10*,*11*). If RacPyV is driving oncogenesis, the transformation location has been remarkably consistent.

In humans, onset of viral-associated cancer typically occurs decades after initial infection with a transforming virus; however, free-ranging raccoons are short lived (≈2–3 years), so this naturally occurring transformation event must be relatively rapid. Natural models of viral-induced tumorigenesis provide an excellent opportunity to analyze mechanisms of transmission, transformation, viral persistence, and routes of shedding and infection. Raccoons are undeniably successful in the human suburban environment and have been proposed as a sentinel species for environmental contaminants and exposure to pathogens (*36*). New animal PyV species have been identified frequently during the last few years, but the strong association of RacPyV with a relatively sudden occurrence of brain tumors distinguishes it from other PyVs, and its discovery could help shed light on the etiologic role of PyVs in oncogenesis.

Technical Appendix Primers used in the identification of raccoon polyomavirus; Southern blot hybridization probe amplification verification; Southern blot hybridization probe and restriction site map; and polyomaviruses used for consensus PCR primer design.

## References

[1] Johne R, Buck CB, Allander T, Atwood WJ, Garcea RL, Imperiale MJ, Taxonomical developments in the family *Polyomaviridae.* Arch Virol. 2011;156:1627–34. 10.1007/s00705-011-1008-x 21562881 PMC3815707

[2] Colegrove KM, Wellehan JF Jr, Rivera R, Moore PF, Gulland FM, Lowenstine LJ, Polyomavirus infection in a free-ranging California sea lion (*Zalophus californianus*) with intestinal T-cell lymphoma. J Vet Diagn Invest. 2010;22:628–32. 10.1177/104063871002200422 20622238

[3] Rollison DE. Epidemiologic studies of polyomaviruses and cancer: previous findings, methodologic challenges and future directions. Adv Exp Med Biol. 2006;577:342–56. 10.1007/0-387-32957-9_24 16626047

[4] Poulin DL, DeCaprio JA. Is there a role for SV40 in human cancer? J Clin Oncol. 2006;24:4356–65. 10.1200/JCO.2005.03.7101 16963733

[5] Chang Y, Moore PS. Merkel cell carcinoma: a virus-induced human cancer. Annu Rev Pathol. 2012;7:123–44. 10.1146/annurev-pathol-011110-130227 21942528 PMC3732449

[6] Imperiale M, Major E. Polyomaviruses. In: Fields BN, Knipe DM, Howley PM, editors. Fields virology, 5th ed. Philadelphia: Lippincott Williams & Wilkins; 2007. p. 2263–98.

[7] Walker DL, Padgett BL, ZuRhein GM, Albert AE, Marsh RF. Human papovavirus (JC): induction of brain tumors in hamsters. Science. 1973;181:674–6. 10.1126/science.181.4100.674 4353360

[8] London WT, Houff SA, Madden DL, Fuccillo DA, Gravell M, Wallen WC, Brain tumors in owl monkeys inoculated with a human polyomavirus (JC virus). Science. 1978;201:1246–9. 10.1126/science.211583 211583

[9] Ohsumi S, Ikehara I, Motoi M, Ogawa K, Nagashima K, Yasui K. Induction of undifferentiated brain tumors in rats by a human polyomavirus (JC virus). Jpn J Cancer Res. 1985;76:429–31 . 2991057

[10] Del Valle L, Gordon J, Ferrante P, Khalili K. JC virus in experimental and clinical brain tumors. In: Khalili K, Stoner G, editors. Human polyoviruses molecular and clinical perspectives. New York: Wiley-Liss, Inc.; 2001. p. 409–30.

[11] Barbanti-Brodano G, Martini F, De Mattei M, Lazzarin L, Corallini A, Tognon M. BK and JC human polyomaviruses and simian virus 40: natural history of infection in humans, experimental oncogenicity, and association with human tumors. Adv Virus Res. 1998;50:69–99. 10.1016/S0065-3527(08)60806-4 9520997

[12] White MK, Gordon J, Reiss K, Del Valle L, Croul S, Giordano A, Human polyomaviruses and brain tumors. Brain Res Brain Res Rev. 2005;50:69–85. 10.1016/j.brainresrev.2005.04.007 15982744

[13] Pipas JM. SV40: cell transformation and tumorigenesis. Virology. 2009;384:294–303. 10.1016/j.virol.2008.11.024 19070883

[14] Lednicky JA, Butel JS. Polyomaviruses and human tumors: a brief review of current concepts and interpretations. Front Biosci. 1999;4:d153–64. 10.2741/Lednicky 9989950

[15] Hamir AN. Pathology of neurologic disorders of raccoons (*Procyon lotor*). J Vet Diagn Invest. 2011;23:873–84. 10.1177/1040638711416851 21908341

[16] Mikaelian I, Martineau D, Helie P, Patenaude R, Campbell D, Barker IK. Tumors in wild adult raccoons from a suburban area. Can Vet J. 1999;40:429–30 . 10367163 PMC1539736

[17] McAloose D, Newton AL. Wildlife cancer: a conservation perspective. Nat Rev Cancer. 2009;9:517–26. 10.1038/nrc2665 19550426 PMC7096862

[18] Del Valle L, Gordon J, Assimakopoulou M, Enam S, Geddes JF, Varakis JN, Detection of JC virus DNA sequences and expression of the viral regulatory protein T-antigen in tumors of the central nervous system. Cancer Res. 2001;61:4287–93 . 11358858

[19] Krynska B, Otte J, Franks R, Khalili K, Croul S. Human ubiquitous JCV(CY) T-antigen gene induces brain tumors in experimental animals. Oncogene. 1999;18:39–46. 10.1038/sj.onc.1202278 9926918

[20] Caracciolo V, Reiss K, Khalili K, De Falco G, Giordano A. Role of the interaction between large T antigen and Rb family members in the oncogenicity of JC virus. Oncogene. 2006;25:5294–301. 10.1038/sj.onc.1209681 16936750

[21] Frisque RJ, Hofstetter C, Tyagarajan SK. Transforming activities of JC virus early proteins. Adv Exp Med Biol. 2006;577:288–309. 10.1007/0-387-32957-9_21 16626044

[22] Reich NC, Levine AJ. Specific interaction of the SV40 T antigen-cellular p53 protein complex with SV40 DNA. Virology. 1982;117:286–90. 10.1016/0042-6822(82)90531-1 6278740

[23] Dyson N, Bernards R, Friend SH, Gooding LR, Hassell JA, Major EO, Large T antigens of many polyomaviruses are able to form complexes with the retinoblastoma protein. J Virol. 1990;64:1353–6 . 2154613 10.1128/jvi.64.3.1353-1356.1990 PMC249255

[24] Del Valle L, Gordon J, Assimakopoulou M, Enam S, Geddes JF, Varakis JN, Detection of JC virus DNA sequences and expression of the viral regulatory protein T-antigen in tumors of the central nervous system. Cancer Res. 2001;61:4287–93 . 11358858

[25] Del Valle L, Pina-Oviedo S, Perez-Liz G, Augelli BJ, Azizi SA, Khalili K, Bone marrow-derived mesenchymal stem cells undergo JCV T-antigen mediated transformation and generate tumors with neuroectodermal characteristics. [Epub ahead of print]. . Cancer Biol Ther. 2010;9. 10.4161/cbt.9.4.10653 20190567 PMC2921558

[26] Pipas JM. Common and unique features of T antigens encoded by the polyomavirus group. J Virol. 1992;66:3979–85 . 1318392 10.1128/jvi.66.7.3979-3985.1992 PMC241200

[27] Sablina AA, Hahn WC. SV40 small T antigen and PP2A phosphatase in cell transformation. Cancer Metastasis Rev. 2008;27:137–46. 10.1007/s10555-008-9116-0 18214640

[28] Deuzing I, Fagrouch Z, Groenewoud MJ, Niphuis H, Kondova I, Bogers W, Detection and characterization of two chimpanzee polyomavirus genotypes from different subspecies. Virol J. 2010;7:347. 10.1186/1743-422X-7-347 21110837 PMC3003640

[29] Shuda M, Kwun HJ, Feng H, Chang Y, Moore PS. Human Merkel cell polyomavirus small T antigen is an oncoprotein targeting the 4E–BP1 translation regulator. J Clin Invest. 2011;121:3623–34. 10.1172/JCI46323 21841310 PMC3163959

[30] Diters RW, Kircher CH, Nielsen SW. Astrocytoma in a raccoon. J Am Vet Med Assoc. 1978;173:1152–3 . 738936

[31] Hamir AN, Picton R, Blythe LL, Heidel JR. Diagnostic exercise: astrocytoma with involvement of medulla oblongata, spinal cord, and spinal nerves in a raccoon (*Procyon lotor*). Vet Pathol. 2008;45:949–51. 10.1354/vp.45-6-949 18984803

[32] Bofill-Mas S, Pina S, Girones R. Documenting the epidemiologic patterns of polyomaviruses in human populations by studying their presence in urban sewage. Appl Environ Microbiol. 2000;66:238–45. 10.1128/AEM.66.1.238-245.2000 10618230 PMC91812

[33] Yu J, Boyapati A, Rundell K. Critical role for SV40 small-t antigen in human cell transformation. Virology. 2001;290:192–8. 10.1006/viro.2001.1204 11883184

[34] zur Hausen H. The search for infectious causes of human cancers: where and why. Virology. 2009;392:1–10. 10.1016/j.virol.2009.06.001 19720205

[35] Carbone M, Pannuti A, Zhang L, Testa JR, Bocchetta M. A novel mechanism of late gene silencing drives SV40 transformation of human mesothelial cells. Cancer Res. 2008;68:9488–96. 10.1158/0008-5472.CAN-08-2332 19010924 PMC2666620

[36] Bigler WJ, Jenkins JH, Cumbie PM, Hoff GL, Prather EC. Wildlife and environmental health: raccoons as indicators of zoonoses and pollutants in southeastern United States. J Am Vet Med Assoc. 1975;167:592–7 . 170239

