# Supplementary material for: Novel Polyomavirus associated with Brain Tumors in Free-Ranging Raccoons, Western United States
Source: Emerg Infect Dis. 2013 Jan;19(1):77–84. doi: 10.3201/eid1901.121078 (PMC3558004; doi:10.3201/eid1901.121078)
Supplement: Technical Appendix — Primers used in the identification of raccoon polyomavirus; Southern blot hybridization probe amplification verification; Southern blot hybridization probe and restriction site map; and polyomaviruses used for consensus PCR primer design. [file 12-1078-Techapp-s1.pdf]

Article DOI: http://dx.doi.org/10.3201/eid1901.121078

# Novel Polyomavirus associated with Brain Tumors in Free-Ranging Raccoons, Western United States

## Technical Appendix

Technical Appendix Table. Primers used in the identification of raccoon polyomavirus

| Name | Sequence (5′ → 3′) |
|---|---|
| PLMA_F3 | GSNGGNGTNGCNTGGTAY |
| PLMA_F5_1of2 | GGNAARACNACNSTDGCN |
| PLMA_F5_2of2 | GGNAARACHACHTTRGCN |
| PLMA_R1 | YTGHCCYTTDACATCYTCAAA |
| PLMA_R4 | NCCTTTHACATCYTCAAAHAC |
| PLMA_R5_1of2 | RTCRATRGCRCAKCCYAGYTCRAA |
| PLMA_R5_2of2 | RTCRATRGCRCAKCCTAAYTCRAA |
| PLMA_LTag01_F | CATGTTGAAGGGACTCCTGCAATTATTTAC |
| PLMA_LTag02_R | AGTTTATCTGCTGGACAGTTGATGTTTAAGG |
| PLMA_F1 | CAYCCNGAYAARGGNGGN |

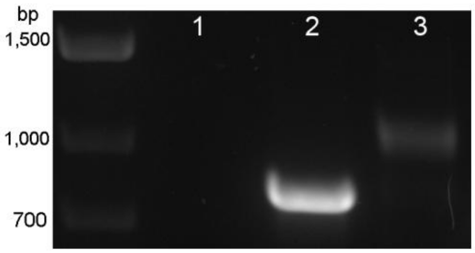


Technical Appendix Figure 1. Southern blot hybridization probe amplification verification. Digoxigenin (DIG)-labeled probe for Southern blot hybridization. Lane 1 is a water-negative control. Lane 2 is amplification of the 799-bp RacPyV_LT_Probe1 with regular dNTPs. Lane 3 is amplification of RacPyV_LT_Probe1 with DIG-dUTP. As expected, the DIG-labeled amplicon runs slower and appears fainter because of high-density DIG-labeling. RacPyV, raccoon polyomavirus.

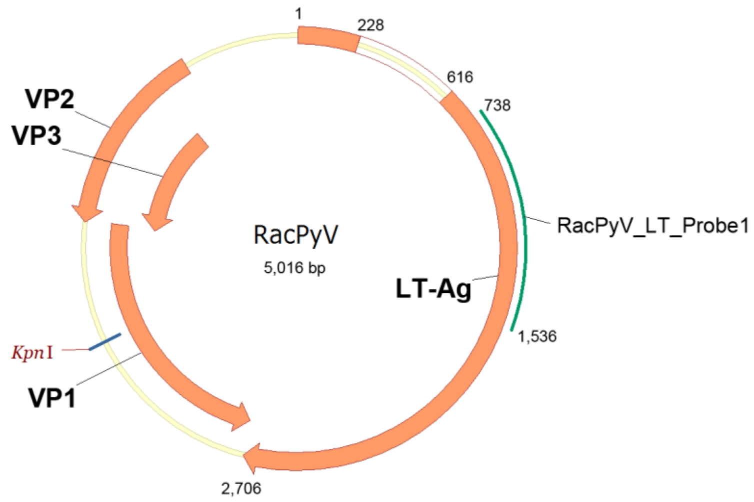


Technical Appendix Figure 2. Southern blot hybridization probe and restriction site map. A highly conserved (≥99.87% identity) 799-bp region of RacPyV LT-Ag spanning nt 738–1,536 of the RacPyV4 genome was chosen as the probe for the Southern blot hybridizations. A unique, conserved *Kpn*I restriction site within VP1 linearizes the RacPyV circular genome. RacPyV, raccoon polyomavirus; LT-Ag, large T-antigen; VP, viral protein.

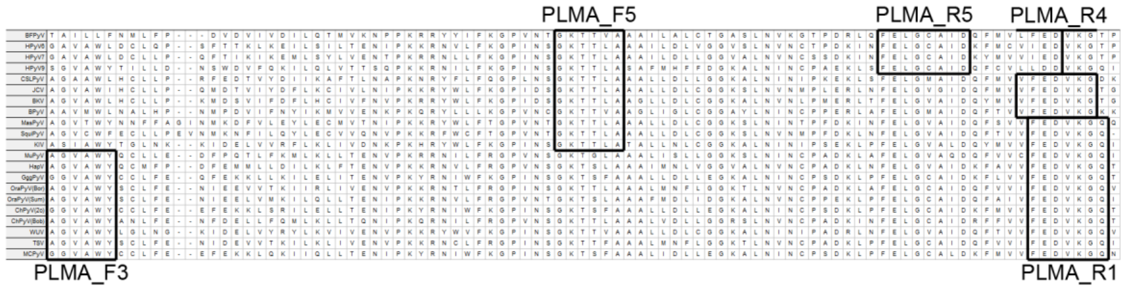


Technical Appendix Figure 3. Polyomaviruses used for consensus PCR primer design. The large T-antigens of 21 polyomaviruses as described in materials and methods were aligned and scanned for conserved regions to generate consensus PCR primers.
